# Applying the net-benefit framework for assessing cost-effectiveness of interventions towards universal health coverage

**DOI:** 10.1186/1478-7547-10-8

**Published:** 2012-07-16

**Authors:** Sennen Hounton, David Newlands

**Affiliations:** 1 Department of Epidemiology, Centre MURAZ, 2054 Avenue Mamadou KONATE, Bobo-Dioulasso, 01 BP 390, Burkina Faso; 2 Business School, University of Aberdeen, Edward Wright Building, Dunbar Street, Old Aberdeen, AB24 3QY, Scotland, United Kingdom

**Keywords:** Cost-effectiveness analysis, Net-benefit framework, Universal health coverage

## Abstract

In assessing the cost-effectiveness of an intervention, the interpretation and handling of uncertainties of the traditional summary measure, the Incremental Cost Effectiveness Ratio (ICER), can be problematic. This is particularly the case with strategies towards universal health coverage in which the decision makers are typically concerned with coverage and equity issues. We explored the feasibility and relative advantages of the net-benefit framework (NBF) (compared to the more traditional Incremental Cost-Effectiveness Ratio, ICER) in presenting results of cost-effectiveness analysis of a community based health insurance (CBHI) scheme in Nouna, a rural district of Burkina Faso. Data were collected from April to December 2007 from Nouna’s longitudinal Demographic Surveillance System on utilization of health services, membership of the CBHI, covariates, and CBHI costs. The incremental cost of a 1 increase in utilization of health services by household members of the CBHI was 433,000 XOF ($1000 approximately). The incremental cost varies significantly by covariates. The probability of the CBHI achieving a 1% increase in utilization of health services, when the ceiling ratio is $1,000, is barely 30% for households in Nouna villages compared to 90% for households in Nouna town. Compared to the ICER, the NBF provides more useful information for policy making.

## Background

**Figure 1:**
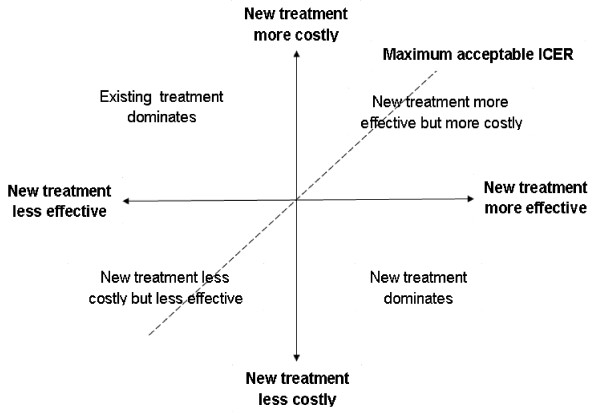
Cost-effectiveness plane.

Economic evaluation in general, and cost-effectiveness analysis in particular is deemed by scholars, health administration and policy experts a centerpiece of the decision making process by balancing health gains against costs of interventions [1]. The results of cost effectiveness analysis provide a rationale to enhance the efficiency of resource allocation. Traditionally, the use of cost effectiveness analysis as a tool to inform and guide resources allocation has revolved around the Incremental Cost Effectiveness Ratio (ICER), which indicates the additional amount of money needed to obtain an extra unit of health gain or to prevent an adverse event compared to alternatives. However, apart from few situations with a clearly dominant intervention (less effective and more costly or more effective and less costly) compared to alternatives, most interventions will present a situation in which there is a need of a threshold value, a ceiling that the decision maker is willing to pay as a good value for money (Figure 1). New interventions in clinical medicine are likely to be more effective and more costly because breakthroughs in medical procedures and new technologies are typically more expensive than existing practices. In these cases, not only is there a need to estimate the maximum a provider (society, or the health system for example) is willing to pay for an additional unit of health gain, but it is also difficult to reliably build confidence intervals around the ICER estimates for inferential analysis. The value of the maximum a provider is willing to pay for an additional unit of health gain is often estimated through extensive willingness to pay surveys and is not always available to the analysts. The use of the net-benefit framework, a recently developed approach and mostly applied in pharmacoeconomics and clinical interventions [2,3], presents the potential to overcome the current limitations of handling of uncertainties and reliance on the maximum value a provider is willing to pay to achieve an extra unit in health gain for the decision rule in public health [4-8]. The aim of this study was to assess the feasibility and relative advantages of the net-benefit framework in assessing the cost-effectiveness of an intervention towards universal health coverage compared to the traditional ICER approach.

## Methods

### Context and participants

**Figure 2:**
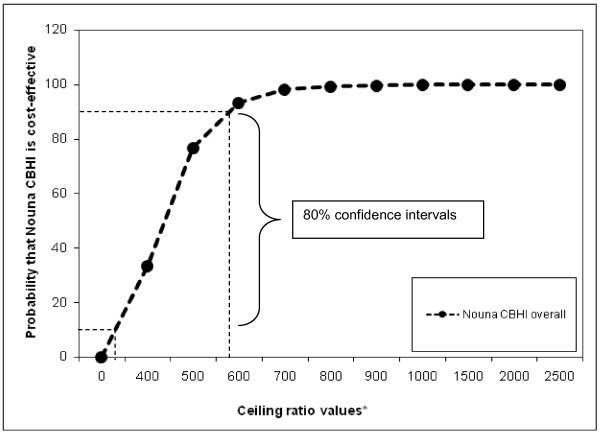
**Cost-effectiveness acceptability curves, Nouna CBHI, Burkina Faso.** * Please note on X-axis actual values correspond to displayed values * 1000.

The Nouna health district, also referred to as Kossi province, is a rural health district situated in the North West of Burkina Faso. The area is characterized by dry weather with a mean annual rainfall of about 800 mm resulting in dry savannah vegetation. In the early 1990s, a Demographic Surveillance System (DSS) was established by the Nouna Health Research Centre. The original DSS area covered 39 villages (~population about 26 000 inhabitants) and has been progressively extended to cover 58 villages and Nouna town, with a population of about 72 000 people. The density of population was about 35 individuals per square km. The population is distributed in roughly 9,500 households and composed of 65% rural dwellers and 35% Nouna semi-urban dwellers. The population is essentially young with children less than 15 years of age representing about 48% of the total population, and only 6.2% above 60 years of age. The inhabitants are mostly subsistence farmers and/or cattle keepers, and illiteracy is extremely high, over 80% (Figure 2).

### The interventions

The Nouna community based health insurance scheme (CBHI) was launched in 2004 and was developed by the Nouna Health Research Centre as an operational research project to study how to improve community access and uptake of health services and how to meet the need of the poor within Nouna health district. The intervention has been extensively described in the literature [9-13]. It is a voluntary community health insurance scheme which aims to reduce financial barriers (out-of-pocket payments) and improve quality of care, thus improving access and uptake of health care. The alternative intervention will be the status quo (no enrolment in the CBHI).

### Data collection and analysis

Data were extracted from the longitudinal Nouna demographic and surveillance site on membership of the Nouna CBHI, utilization of health services, the average distance from village to health centre, assets ownership, age and education level of the head of household. In order to generate household-level costs of the CBHI scheme from a societal perspective we added, for every household member of the scheme, the household costs (enrolment fees and premium) to the average cost of enrolling in the Nouna CBHI scheme from the health system perspective. The latter was obtained by dividing the 2007 annual costs of running the Nouna CBHI scheme by the number of households, members (370) in 2007. For households which are not members of the scheme, there is obviously no cost incurred for membership fees. However, these households have to meet the cost of the utilization of health services out of pocket. We use the estimated cost of the benefit package of the Nouna CBHI which was 9630 West Africa francs (XOF), equivalent to approximately $20) in 2004 [12]. We computed ICER for extra additional utilization of health services, and cost effectiveness acceptability curves (CEAC) to illustrate the decision rule of cost effectiveness of the intervention.

The ICER decision rule is that if the estimated ICER lies below the ceiling ratio, which represents the maximum decision makers are willing to pay for an incremental unit of the measure of effectiveness, then the intervention concerned is deemed cost-effective. By varying the ceiling ratio, the varying probability that the intervention is cost-effective can be identified. The CEAC shows the probability of the intervention being cost-effective for all potential values of the ceiling ratio. Unlike the ICER approach, the CEAC can also be employed to obtain a confidence interval of cost-effectiveness. It also avoids the problems of interpretation of a negative ICER [14,15]. There isa straightforward graphical representation and interpretation that a new treatment is not cost effective [7]. In this paper, the likelihood values that the Nouna CBHI is cost-effective compared to the status quo were obtained using the p-values on the Nouna CBHI dummy when running an Ordinary Least Square (OLS) regression. The p-values are 2-sided p-values; however only one-sided probability is needed to test whether the incremental net-benefit is positive (Nouna CBHI is preferred) or negative (the status quo is preferred). The one–sided p-values were thus obtained by dividing the 2-sided p-values by 2. For negative incremental net-benefits the probabilities that Nouna CBHI is preferred equals the one sided p-values, and for positive incremental net-benefits, the probabilities that the Nouna CBHI is preferred equals 1 minus the one sided p-values (Hoch JS et al, 2006). A major strength of this technique when it comes to resource allocation is that for a given budget, one can model the different probabilities that the Nouna CBHI is preferred to the status quo.

However, whilst this technique could be sufficient in clinical care decisions about the choice of preferred medication, technology, or screening exam, in the public health field, a decision maker is often concerned about issues beyond the optimality of one intervention over another, especially with issues of equity. This is where the net-benefit framework could potentially be very useful in assessing the effect of significant determinants on the marginal cost-effectiveness of a universal health coverage intervention such as the Nouna community based health insurance.

Given access to health care is influenced by major determinants (such as the distance to health facilities, education, or assets ownership), a net-benefit framework, applied to the cost effectiveness of the Nouna CBHI, could be effective through the joint probability distribution in identifying the most important determinants that affect the cost-effectiveness results. The net-benefit framework employs linear regression techniques, and to date, has been most often used alongside clinical trials of health care regimens or technology devices [2-5]. Thus, it has the potential even for observational studies with patient-level effect and cost data, for the better presentation and interpretation of cost-effectiveness results and better evidence based decision making.

The traditional equation ΔC/ΔE (ICER) can be re-arranged by multiplying each arm of the equation by ΔE. The result is ΔC = ΔE * ICER and for any ceiling ratio Ro, ΔC = ΔE * Ro. Thus, a net-benefit statistic can be computed as follows: ΔE * Ro − ΔC = ΔNB. We computed for each observation (household) in the household survey an individual net-benefit statistic. The expression of an individual net-benefit NBi = ΔEi * Ro − ΔCi is similar to a traditional linear regression equation Y = α + δX_i_ + ε_i_ where Y is the dependent variable, α is the intercept, δ the coefficient on an explanatory variable (continuous variable or dummy variable taking the value 1 for a positive outcome and 0 for a negative outcome for example) and ε_i_ is the standard error. Thus, for the Nouna community based health insurance scheme, the household net-benefit could be modeled as NB_i_ = α + δCBHI_i_ + ε_*i*_ where NB_i_ is the net-benefit for each subject (or household), α is the intercept, CBHI_i,_ is the intervention (taking the value zero if a household is not a member of the scheme and 1 for a member), δt_i,_ is the incremental net benefit and ε_i_ is the standard error. The interpretation is straightforward and when this difference is greater than zero, it means that the incremental cost for one additional unit of effectiveness (in this case utilization of health services) is below the Ro (the maximum the provider is willing to pay). The CBHI will be deemed cost-effective in relation to the status quo. Similarly, if the coefficient is negative, then the incremental cost for one additional unit of effectiveness is above the Ro and the status quo will be deemed cost-effective.

The basic model above (NB_i_ = α + δCBHI_i_ + ε_i_) could then be expanded to include important covariates and thereby allow the examination of the marginal impact of these covariates on incremental cost effectiveness. The final model may look like: NB_i_ = α + ∑_j=1_^P^ β_j_ x_ij_ + δt_i_ + ∑_j=1_^P^ ý_j_ x_ij_ + ϵ_i_ where NB_i_ is the summation of the interaction between the treatment dummy (Community Based Health Insurance for example, coded yes or no) and the covariates. ý’s magnitude and significance indicates how the cost effectiveness of CBHI is expected to vary at the margin. Thus the use of the net-benefit model for presenting and interpreting cost-effectiveness analysis results has the potential to overcome the double dilemma of not being able to access progress using outcomes measures (for example, computing maternal or perinatal mortality) and not being able to reliably assess cost-effectiveness using incremental cost-effectiveness ratios. As indicated in the background section, cost effectiveness analysis traditionally relies on use of an incremental cost effectiveness ratio (ICER) to indicate, among a set of alternative strategies, which is the most cost effective. Not only does the ICER as a ratio not indicate what to do, how to do it or where to do it, the decision rule is not straightforward when there is no clear dominance of one alternative over another [2,6,14]. Moreover, there are very few situations in which decision makers decide to solely go with one strategy over another. Rather, they are more likely to allocate resources across a range of complementary strategies for maximum health gains and thus the net-benefit framework offers an advantage over the traditional ICER approach in presenting and interpreting results for public health interventions (Table 1).

**Table 1:** Relative advantages of net-benefit framework and incremental cost-effectiveness ratio for presenting and interpreting results of cost-effectiveness analysis

**Relative advantage criteria**	**Standard Incremental Cost Effectiveness Ratio (ICER)**	**Net Benefit Framework**
Type of analysis	Descriptive analysis, and stratified analysis (by important covariates)	Regression analysis, and joint probability distribution with important covariates
Confidence interval	No	Yes
Requirement of contextually relevant threshold (ceiling ratio)	Yes, to assess if intervention is good value for money	No, hypothetical ceiling ratios can be plotted and probabilities of cost effectiveness calculated
Adjustment to covariates (important sub groups)	No	Yes
Variability explained by covariates	No	Yes
Relative advantages for interpretation	Simple point estimate, greater or lower than a ceiling ratio	Graphical presentation; illustration of alternative scenarios with different ceiling ratios

### Ethical consideration

The study was approved by the ethical review board of Nouna Health Research Centre.

## Results

### Descriptive analysis of the study populations

Table 2 describes the characteristics of the households included in the Nouna panel household survey by enrolment status in the Nouna CBHI scheme and by the selected covariates (education, place, perceived quality of care, asset ownership). The two groups are comparable with respect to mean age of head of household (49.6 for non-members versus 50.8 for households members, t-test p = 0.148). There were significant differentials in enrolment in the Nouna CBHI scheme by utilization of health services and by covariates. There was a 14 percentage point difference (85.4 - 71.4) in the utilization of health services between members and non-members. Similarly there were 20.6 (59.3 – 38.7), 23.1 (63.2 – 40.1), and 18.7 (34.3 – 15.6) percentage point differences between members and non-members for people with at least primary level of education, people living in Nouna town, and assets ownership, respectively.

**Table 2:** Descriptive characteristics of populations by enrolment status (from household survey, 1504 household, 2007)

**Characteristics**	**Members**	**Not members**	**P-value**
	**N (%)**	**N (%)**	
**Use of health services**			0.000
- Did not use	53 (14.6)	281 (28.6)	
- Have used	310 (85.4)	700 (71.4)	
**Education**			0.000
- None	148 (40.7)	602 (61.3)	
- At least primary school level	216 (59.3)	380 (38.7)	
**Place**			0.000
- Nouna town	230 (63.2)	394 (40.1)	
- Nouna villages	134 (36.8)	588 (59.9)	
**Asset ownership**			0.000
- Most poor	3 (0.8)	235 (23.9)	
- Second quartile	35 (9.6)	235 (23.9)	
- Third quintile	85 (23.4)	198 (20.2)	
- Fourth quintile	116 (31.9)	161 (16.4)	
- Least poor	125 (34.3)	153 (15.6)	

P-values are the level of significance on the Pearson chi-square test between enrolment and covariates. There is no difference by age or sex between members and not members.

### Standard cost-effectiveness analysis

In Table 3, the Incremental Cost Effectiveness Ratio (ICER) was obtained by dividing the difference in average cost between the intervention and comparison groups (70.253 – 9630) by the difference in average effect (utilization of health services) between the intervention and comparison groups (0.85 – 0.71). The result is equal to 433,000 XOF (approximately $1000) and is interpreted as the overall incremental in cost for achieving one additional increase in household’s utilization of health services (unit of effectiveness) for households members in the community based insurance scheme compared to household non-members. At this point the Nouna CBHI scheme appears to be more costly and more effective. The ICER estimates vary significantly by covariates. For example, the incremental cost for one extra institutional delivery within Nouna villages was 530,600 XOF (approximately $1250) compared to 301,700 XOF (approximately $700) in Nouna town. The stratified ICER results (only place of residence reported in this paper) confirm the existence of important subgroups and prompt interest in assessing how these covariates affect the overall cost-effectiveness of the intervention.

**Table 3:** Sample statistics from the economic evaluation of the Nouna CBHI, data with net-benefit, household survey, 2007, Nouna districts Burkina Faso

			
**Group variables**	**Mean**	**SD**	**SE**
**Overall analysis**			
*Comparison group*			
*(Not members N = 982 )*			
Cost	9630	0.000	0.000
Effect (%)	71	0.452	0.014
*Intervention group*			
*(Members N = 364)*			
Cost	70253	11658	611
Effect (%)	85	0.125	0.019
*Increments*			
Cost difference*	60623	-	372
Effect difference (%)	14		
**Sample ICER**	**4330**		
**Place of residence**			
*Nouna villages group*			
Cost difference	64207	-	545
(%) Effect difference	12.1	-	0.039
Sample ICER	**5306**		
*Nouna town group*			
Cost difference	58535.5	-	507
(%) Effect difference	19.4	-	0.036
Sample ICER	**3017**		
**Incremental net-benefit**	Coefficient (SE)		
Values of ceiling ratio (R)	Overall	Nouna town	Nouna villages
*R = 0*	- **60623** (193)	- **58535.5** (507)	- **64207** (545)
*R = 500 000*	9577 (13143)	38324 (18335)	- 3806 (19683)
*R = 700 000*	28034 (18400)	77075 (25667)	20354 (27543)
*R = 1 000 000*	70165 (26286)	135203 (36692)	56594 (39335)

ICER = Cost of having one additional% of utilization of health service among members.

* Equal variance not assumed. The costs are in CFA, West Africa French currency.

### Applying the net-benefit regression approach

Table 3 presents the results of the overall economic evaluation with a range of arbitrary ceiling ratios (arbitrary but selected around the sample ICER). We use place of residence (Nouna town versus Nouna villages) as examples of covariates and present net-benefits estimates for different values of the ceiling ratio (Ro) including zero. The coefficients were obtained from an Ordinary Least Squares (OLS) regression as explained above and correspond to the increment net-benefit. The aim of this analysis and the results displayed in this table is to demonstrate that the standard descriptive analysis for computing an Incremental Cost-Effectiveness Ratio (ICER) is equivalent to the net-benefit framework, as the linearization of the equation ICER = λ. One can verify that when λ is = 0, the increment net-benefit (nb2 - nb1) = λ*(Average effect1 – Average effect1) – (Average cost2 - Average cost1) = 0*(85 – 71) – (70253 – 9630) = − 60623.

For example, when the ceiling value is 700,000 XOF (equivalent to $1600) for example, the coefficients obtained from the OLS regression for the overall sample, populations in Nouna town, and populations in Nouna villages are 37664 XOF, 77075 XOF, and 20354 XOF respectively. It can be verified manually that these numbers correspond (apart from rounding errors) to what would have been obtained by the equation (nb) = λ* (effect) – (cost), where (nb) is the average net-benefit, 700,000 the ceiling ratio (λ), (effect) is the mean effect and (cost) is the mean cost.

Table 4 presents the variations of the incremental net benefit of the intervention with different ceiling ratios and the probability of cost-effectiveness of Nouna CBHI with different ceiling ratios. The probabilities that the CBHI is cost-effective were computed and used to construct the cost-effectiveness acceptability curve (Figure 2).

**Table 4:** Simple net-benefit regression estimates with different ceiling ratios, Nouna community based health insurance, household survey, 2007, Burkina Faso

**N = 1344**	**NMB With**	**NMB With**	**NMB With**	**NMB With**	**NMB With**	**NMB With**
	**λ=0**^**a**^	**λ=500000**	**λ=700000**	**λ=1000000**	**λ=1500000**	**λ=2000000**
**Explanatory**	**[SE] (p-value)**	**[SE] (p-value)**	**[SE] (p-value)**	**[SE] (p-value)**	**[SE] (p-value)**	**[SE] (p-value)**
Variables						
Constant term	9630	347148	489860	703927	1060706	1417485
	[193] (1.000)	[6830] (0.000)	[9562] (0.000)	[13660] (0.000)	[193] (0.000)	[193] (0.000)
						220232
Intervention strategy(CBHI)	- 60623	9577	37664	79795	150013	[372] (0.000)
	[372] (0.000)	[13143] (0.466)	[18400] (0.041)	[26286] (0.002)	[372] (0.000)	
R^2^ (adjusted)	0.952	0.000	0.003	0.007	0.011	0.013
F (1, 1344)	26587	0.531	4.2	9.2	14.4	17.5
Prob > F	< 0.000	< 0.000	< 0.000	< 0.000	< 0.000	< 0.000

^a^ When **λ** =0, NMB = − Cost. The values of **λ** = are in CFA, West Africa French currency.

### Cost effectiveness acceptability curves

The cost-effectiveness acceptability curve graphically represents the levels of certainty around the cost-effectiveness analysis ratio of two interventions by plotting hypothetical estimates of ceiling ratios against the probability that a new intervention (the Nouna CBHI scheme in our case) is preferred over an existing intervention (the status quo). As can be seen on Figure 2 if the decision makers in Nouna are only prepared to pay a ceiling ratio less than 400,000 XOF ($920) the probability that the Nouna CBHI scheme is preferred or deemed cost effective is small less than 30%. However, if decision makers in Nouna are willing to pay a ceiling ratio more than 700,000 XOF ($1600) the probability that the Nouna CBHI scheme is preferred or deemed cost effective increases to above 99% (asymptotic and close to but never reaching 100%).

**Figure 3:**
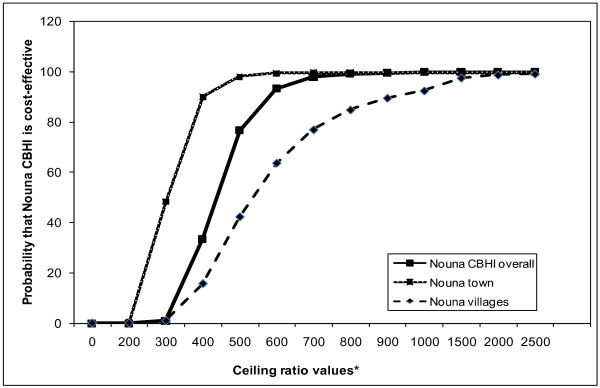
**Unadjusted cost-effectiveness acceptability curves, Nouna CBHI overall and with covariate place, Burkina Faso.** * Please note on X-axis actual values correspond to displayed values * 1000.

The same calculations and graphical representations performed for the Nouna CBHI (Tables 4–5, Figure 2) could be computed for subgroups of the sample by important covariates such as place of residence (dummy variable coded 0 for Nouna villages and 1 for Nouna town) or education (dummy variable coded 0 for no education and 1 for some education). The results are presented in Table 6 and Figures 3. Overall, lower ceiling ratios are needed to achieve a given probabilities that the Nouna CBHI is cost-effective in Nouna town versus Nouna villages. In the communities of Nouna villages, the probability that the Nouna CBHI scheme is cost-effective is close to zero when the decision makers in Nouna are only prepared to pay less than 300,000 XOF ($690) for one extra use of health services and only approaches 100% above a ceiling ratio of 1,500,000 XOF ($3460. However, in Nouna town the probability that the Nouna CBHI scheme is cost-effective is nearly 50% at the ceiling ratio of 300,000 ($690) for one extra use of health services and approaches 100% at a ceiling ratio about 500,000 XOF ($1150).

**Table 5:** Cost-effectiveness acceptability curves from the net-benefit regression, Nouna CBHI, household survey, 2007, Burkina Faso

**Values of ceiling ratio**	**Treatment (intervention) coefficients**		**One sided p-value**	**Probability of cost-effectiveness (OLS regression)**
	**Estimates**	**P-values**		**%**
0	- 60623	0	0	0
400 000	- 4466	0.671	0.335	33.5
500 000	9577	0.466	0.233	76.7
600 000	23620	0.134	0.067	93.3
700 000	37664	0.041	0.020	98
800 000	51708	0.014	0.007	99.3
900 000	65751	0.006	0.003	99.7
1 000 000	79795	0.002	0.001	99.9
1 500 000	150013	< 0.000	< 0.000	< 100
2 000 000	220232	< 0.000	< 0.000	< 100
2 500 000	290450	< 0.000	< 0.000	< 100

The values of ceiling ratio are in CFA, West Africa French currency.

**Table 6:** Cost-effectiveness acceptability curves, net-benefit OLS regression, Nouna CBHI (Place covariate)

**Nouna Town**					**Nouna villages**			
**Values of ceiling ratio**	**Treatment (intervention) coefficients**		**One sided p-value**	**Probability of cost-effectiveness**	**Treatment (intervention) coefficients**		**One sided p-value**	**Probability of cost-effectiveness**
	**Estimates**	**P-values**		**%**	**Estimates**	**P-values**		**%**
0	- 58535	0	0	0	- 64207	0	0	0
200 000	−19803	0.007	0.003	0.3	- 40046	0	0	0
300 000	- 427	0.969	0.484	48.4	- 27966	0.018	0.009	0.9
400 000	18948	0.197	0.098	90.2	- 15886	0.314	0.157	15.7
500 000	38324	0.037	0.018	98.2	−3806	0.847	0.423	42.3
600 000	57700	0.009	0.004	99.6	8274	0.726	0.363	63.7
700 000	77075	0.003	0.001	99.9	20353	0.460	0.230	77
800 000	96451	0.001	0.000	99.99	32434	0.303	0.151	84.9
900 000	115827	0.000	0.000	99.99	44514	0.209	0.104	89.6
1 000 000	135203	0.000	0.000	99.99	56594	0.151	0.075	92.5
1 500 000	232081	0.000	0.000	99.99	116995	0.048	0.024	97.6
2 000 000	328960	0.000	0.000	99.99	177395	0.024	0.012	98.8
2 500 000	425840	0.000	0.000	99.99	237796	0.016	0.008	99.2

The values of ceiling ratio are in CFA, West Africa French currency.

**Figure 4:**
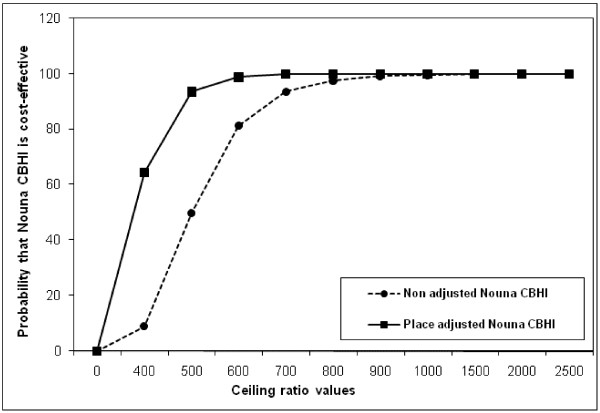
Adjusted (to place of residence) and unadjusted cost-effectiveness acceptability curves, Nouna CBHI, 2007, Burkina Faso. * Please note on X-axis actual values correspond to displayed values * 1000.

We could then assess, as for any regression analysis, the effect of covariates on the cost-effectiveness, the interaction between covariates, any collinearity or residuals. The full results are not presented here as the goal is primarily to demonstrate the applicability of the net-benefit framework to observational public health studies rather than the robustness of the results or any model. We present in Table 7 and on Figure 4 covariates adjusted net-benefit regression estimates with different ceiling ratios, adjusted (to place of residence) and unadjusted cost-effectiveness acceptability curves of the Nouna CBHI respectively. It can be observed in Figure 4 that when adjusted to place (Nouna town versus Nouna villages), the ceiling ratio that corresponds to a 50% probability that the intervention is cost-effective (ICER) is significantly lower than the ICER computed. This is critically important and indicates the importance of adjusting the cost-effectiveness results to known significant covariates. Also, the probability that the Nouna CBHI scheme is cost-effective is higher at any ceiling ratio lower than or equals to 800,000 XOF ($1850) when the net-benefits are adjusted to place of residence (Nouna town versus Nouna villages). This result demonstrates that the probability that the intervention is cost-effective is very high in Nouna town compared to Nouna villages if decision makers in Nouna are willing to pay a ceiling ratio lower than 800,000 XOF ($1850). This important result is more informative than the stratified analysis of the ICER and would not have been possible without a joint probability distribution of the intervention effects and covariates, hence the importance of the net-benefit approach in identifying the effects of important covariates on the cost-effectiveness results.

**Table 7:** Simple net-benefit regression estimates with different ceiling ratios, and covariates adjusted net-benefit regression estimates, Nouna community based health insurance, household survey, 2007, Burkina Faso

**N = 1344**	**NMB With**	**NMB With**	**NMB With**	**NMB With**	**NMB With**	**NMB With**
	**λ =0**^**a**^	**λ =500000**	**λ =700000**	**λ =1000000**	**λ =1500000**	**λ =2000000**
**Explanatory**	**[SE] (p-value)**	**[SE] (p-value)**	**[SE] (p-value)**	**[SE] (p-value)**	**[SE] (p-value)**	**[SE] (p-value)**
Variables						
Constant term	−8595	317202	447519	642993	968784	1294575
	[394] (0.000)	[14159] (0.000)	[19820] (0.000)	[28311] (0.00)	[42466] (0.000)	[56620] (0.000)
**Covariates**						
Education	1700	44652	61835	87609	130565	173522
	[335] (0.000)	[12017] (0.000)	[16821] (0.000)	[24028] (0.000)	[36040] (0.000)	[48053] (0.000)
Place of residence	2255	- 67742	- 95741	- 137739	- 207735	−277731
	[358] (0.000)	[12864] (0.000)	[18007] (0.000)	[25772] (0.000)	[38582] (0.000)	[51443] (0.000)
Asset ownership	- 942	14455	20614	29853	45252	60651
	[136] (0.000)	[4879] (0.003)	[6830] (0.003)	[9756] (0.002)	[14633] (0.002)	[19511] (0.002)
**Intervention (CBHI)**	- 60425	- 10044	23594	59608	119630	179652
	[390] (0.000)	[13995] (0.473)	[19990] (0.229)	[27983] (0.033)	[41973] (0.004)	[55963] (0.001)
R^2^ (adjusted)						
F (4 1343)	0.955	0.031	0.034	0.037	0.041	0.043
Prob > F	7077	10.6	11.6	12.9	14.4	15.2
	0.000	0.000	0.000	0.000	0.000	0.000

^a^ When **λ** =0, NMB = − Cost. The values of ceiling ratio are in CFA, West Africa French currency.

## Discussion

With the traditional ICER, we concluded that approximately $1000 was the incremental in cost for achieving one additional increase in utilization of health services in the intervention area. However, there was no context specific threshold (ceiling ratio) to indicate if the $1,000 was a good value for money for achieving one households’ extra utilization of health services. This estimate provides no clue for what to do, how to do it or where to do it if one is concerned with equity issues in universal health coverage. What could be more helpful is to have some insights in how the cost-effectiveness vary by some equity determinants (for example covering Nouna villages or Nouna town, or design the intervention by groups of households’ assets ownership) which will be the basis for policy making. By applying the net-benefit framework, we were able to conclude that the probability (adjusting for place) of the Nouna community based health insurance to achieve one extra utilization of health services when the ceiling ratio is approximately $1,000 is barely 30% for Nouna villages whilst the corresponding probability for households living in Nouna town is over 90%. This piece of information has important implication for policy making if decision makers are concerned with achieving high probability of cost-effectiveness of the intervention in the poorest population (Nouna villages) and would not have been possible just by using stratified analysis on the traditional ICER approach. As pointed by Hosh JS [3] the existence of important sub-groups affects how the cost-effectiveness varies at the margin and need to be accounted for when analyzing and interpreting cost-effectiveness results. This is the case with universal health coverage.

Identifying what intervention is cost-effective compared to an alternative is an important piece of information, and we have now seen that even if one does not know the true value of the ceiling ratio, using a cost-effectiveness acceptability curve (Figure 2) it is possible to show the level of uncertainty surrounding the estimated ICER. Computing the ICER point estimate (assuming we know the context specific ceiling ratio to rule if a good value for money) does not provide clues about what policy makers could do, how to do it or where to do it when there are important subgroups. Although it is possible to use modeling such as bootstrap method with appropriate weighting [16] to assess how the covariates affect the cost-effectiveness at the margin, modeling will rely on data from secondary sources, instead of actual household level effect and cost data.

If the decision makers are concerned with meeting the needs of the poorest and equity [9,17,18] we argue we need a joint probability distribution of context specific data on cost and effect and the intervention with important determinants of poverty so that policy making be based on the most important determinants (adjusting to known covariates). For example, when examining the net-benefit regression results (Table 7) it can be noted that only a small fraction (3.4%) of the variability in cost-effectiveness can be explained by covariates used in the analysis. This is very small and points to the existence of unknown important variables not captured in our analysis or the low content validity of our constructed metrics. In any case, this type of information is very useful for appraising the likelihood of covariates to affect the cost-effectiveness of the intervention. Although decision making does not revolve around cost-effectiveness analysis results, this type of information may be very useful for policy making and is not possible to have with the more traditional ICER approach. This is the main potential of the net-benefit framework when applying to public health interventions such as universal health coverage.

Lastly, while the main concern of this paper is with methodological challenges, the high values of the ceiling ratios that we found for the CHBI scheme to be cost-effective are of interest. Together with other evidence, such as the lack of any significant reduction in mortality rates, the low enrolment rates [9,10], and the high costs of the scheme [12] cast great doubt upon its cost-effectiveness. Our findings thus challenge the currently fashionable assumption that community based health insurance schemes are a very promising way to extend access to health care in low and middle income countries.

From a methodological point of view, the net –benefit approach opens up the possibility of a marriage of epidemiological, demographic and econometric analytical frameworks in appraising the monetary values of public health interventions. Its application requires the availability of person-level or household-level effect and cost data collection, which in turn will require change to current well established tools and methods of national or sub national household surveys. Although this work was focused on using some of the properties of regression techniques for utilization of health services, there is a possibility, with increasing emphasis on valuing health outcomes in the developing world, to actually use the full potential of regression frameworks and predict for example the net-gains of interventions in saving the lives of women and newborns.. Given this paper is mainly to demonstrate feasibility and applicability of the net-benefit approach and data implications we did not extend on other model diagnosis tests such as normality of residuals.

## Conclusions

There are some challenges in interpreting the traditional ICER results. Regardless of the accuracy of the costing methodology, there remain issues about the level of certainty of the computed estimate. The decision rule of the ICER estimate requires knowledge as to whether the estimate is below or above an externally set value which is the maximum decision makers will be willing to pay for an extra unit of health gain. This value is unknown in most cases but in these circumstances, the net-benefit approach has proven feasible and more insightful in assessing cost-effectiveness of a public health intervention aiming at universal health coverage. The net-benefit approach has the relative advantages of better presentation and interpretation of cost-effectiveness analysis results. The most important advantage in our view is the possible information on the marginal cost-effectiveness of important covariates particularly when decision makers are concerned with important determinants or equity as is often the case in public health interventions. However, its applicability requires appropriate data sets (household-level effect and cost data) which will require that we revise the traditional methods and tools of household surveys to ensure concurrent collection of household-level effect and cost data.

## Competing interests

The authors declare that they have no competing interests

## Authors’ contribution

SH conceived the study (as part of his doctoral thesis), led the data collection, constructed the database, performed the statistical analysis and drafted the manuscript. David Newlands participated in the design of the study, data collection and data analysis. All authors read and approved the final manuscript.

## Authors’ information

Sennen Hounton is a medical epidemiologist with expertise in maternal and newborn health, health systems and economic evaluation. Sennen Hounton is has an MD (Benin), MPH in Epidemiology (University of Oklahoma, USA) and a PhD in Public Health from University of Aberdeen, (Scotland, UK). He was a Senior Research Fellow with Immpact (Initiative for Maternal Mortality Program Assessment). He is currently Maternal Health Technical Adviser at the United Nations Population Fund (New–York), and served as Scientific and Technical Advisor on the WHO Alliance for Health Policy and System Research Scientific and Technical Advisory Committee.

David Newlands is a Senior Lecturer in Economics, previously Team Leader of the Economic Outcomes of an international research programme – Immpact. He is currently a Senior Lecturer at the Business School of University of Aberdeen, Scotland, UK.
